# Human Q fever outbreak investigation and management in six European countries: a One Health compendium of practices and recommendations

**DOI:** 10.1016/j.cmicom.2026.105195

**Published:** 2026-07

**Authors:** Ana Hurtado, Elodie Rousset, Aurélie Couesnon, Tara de Haan, Frederika Dijkstra, Silke F. Fischer, Pierre-Edouard Fournier, Ana L. García-Pérez, Isabel Jado, Elsa Jourdain, Katja Mertens-Scholz, Tom N. McNeilly, Susan Neale, Jane C. Osborne, Marjan Van Esbroeck, Marcella Mori, René van den Brom

**Affiliations:** 1 Animal Health Department, NEIKER—Basque Institute for Agricultural Research and Development, Basque Research and Technology Alliance (BRTA), Derio, Bizkaia, Spain; 2 ANSES, Laboratoire de Sophia Antipolis, Unité fièvre Q animale, Sophia Antipolis, France; 3 Small Ruminant Health Department, Royal GD, Deventer, The Netherlands; 4 Centre for Infectious Disease Control, Netherlands Institute of Public Health and the Environment (RIVM), Bilthoven, The Netherlands; 5 Q fever Consulting Laboratory, Baden-Württemberg State Health Office, Stuttgart, Germany; 6 French Reference Centre for Rickettsioses, Q Fever and Bartonelloses, IHU Méditerranée Infection, Marseille, France; 7 Reference and Research Laboratory on Special Pathogens, National Centre for Microbiology, Instituto de Salud Carlos III (CNM-ISCIII), Madrid, Spain; 8 INRAE, VetAgro Sup, UMR EPIA, University of Clermont Auvergne, Saint-Genès Champanelle, France; 9 Friedrich-Loeffler-Institut, Institute of Bacterial Infections and Zoonoses, Jena, Germany; 10 Moredun Research Institute, Penicuik, UK; 11 Penrith Veterinary Investigation Centre, Animal and Plant Health Agency (APHA), Cumbria, UK; 12 Rare and Imported Pathogens Laboratory, UK Health Security Agency, Porton Down, UK; 13 Department of Clinical Sciences, Institute of Tropical Medicine, Antwerp, Belgium; 14 Sciensano, Belgian Institute for Health, Brussels, Belgium

**Keywords:** Coxiellosis, *Coxiella burnetii*, One Health, Outbreak, Q fever, Ruminants

## Abstract

**Background:**

Q fever is a zoonotic disease caused by *Coxiella burnetii*, with domestic ruminants serving as the main reservoir. Infected animals can shed large quantities of the pathogen into the environment after normal parturition or abortion, enabling airborne transmission to humans and potentially triggering outbreaks of variable scale. Effective outbreak management requires the rapid identification of all related cases, tracing of infection source(s), and implementing proportionate control measures that protect human health while considering animal health and ecosystem integrity.

**Objectives:**

This report aimed to review and compare human Q fever outbreak management protocols across several European countries, identify areas of convergence, highlight operational challenges, and outline key gaps in diagnostic tools, source investigations, and cross-sector coordination. It also seeks to propose research priorities and recommendations to support more coordinated One Health preparedness and response.

**Sources:**

The analysis was conducted within the European Union–funded Q‑Net‑Assess project, involving partners from Belgium, France, Germany, Spain, The Netherlands, and the United Kingdom. Information was drawn from national procedures, guideline documents, and operational experiences shared by researchers and experts affiliated with academic, public health, veterinary, and reference laboratories.

**Content:**

This report describes practices related to outbreak detection, notification, case investigation, identification of sources, and implementation of control measures across countries. It maps key differences in national protocols, highlighting variability in diagnostic approaches, environmental sampling, molecular typing, and cross-sector coordination. The synthesis identifies both shared methodological elements and structural limitations that affect timely and effective outbreak management.

**Implications:**

Findings underscore the need for more harmonized and integrated Q fever outbreak management across Europe. The report outlines research priorities and provides recommendations aimed at strengthening diagnostic capacity, enhancing environmental and molecular investigation tools, and improving One Health collaboration. These insights have potential relevance for other airborne zoonotic diseases.

## Introduction

Q fever (or coxiellosis in animals) is a globally distributed zoonotic disease caused by *Coxiella burnetii*, with domestic ruminants acting as the main reservoirs [1,2]. Infected animals often remain asymptomatic but can abort and shed the bacterium, particularly around parturition [[3], [4], [5], [6]], causing heterogeneous environmental contamination [4]. *C. burnetii* alternates between an intracellular replicating large cell variant and a metabolically weakly active or dormant small cell variant, passing through a transition state cell variant [7,8]. Putative extracellular survival forms detected in the environment may correspond to small cell variants [[7], [8], [9]], but their resistance in the environment [5,[10], [11], [12], [13], [14]] is insufficiently characterized.

Human infection occurs primarily through inhalation of contaminated aerosols, although airborne dissemination pathways remain poorly understood. Person-to-person transmission of *C. burnetii* is uncommon, with a single report of transmission via blood transfusion [15] and rare documented cases linked to organ transplantation [16,17], sexual transmission [18], or during obstetric care [19]. The incubation period typically ranges from 14 to 21 days but can vary widely with infective dose, strain, exposure route, and host-related factors (e.g. gender, age, and condition of the patient). Early infection is detectable by PCR during short-transient bacteraemia, whereas antibodies become detectable from day 5 to 14 onwards [20]. *C. burnetii* presents two antigenic phases (I and II) [21], which form the basis of serological testing to differentiate acute from chronic Q fever (Note S1).

Approximately 60% of human *C. burnetii* infections are asymptomatic, while symptomatic patients typically present with mild flu-like symptoms [22,23] occasionally progressing to pneumonia, febrile acute hepatitis, or meningoencephalitis. Although most infections resolve, 1–5% evolve into chronic, potentially fatal forms (notably vascular infection or endocarditis) [24]. Chronic fatigue syndrome has also been reported after acute Q fever [25]. Clinical presentations vary geographically (e.g. pneumonia predominates in northern Spain and hepatitis in southern regions and the Canary Islands [26]) likely reflecting the marked genetic diversity of circulating *C. burnetii* strains [27,28]. The broad clinical spectrum, combined with the absence of pathognomonic signs and reliance on laboratory confirmation, challenges diagnosis and may contribute to substantial under-recognition.

Most acute Q fever cases respond well to early doxycycline treatment, whereas alternative antibiotics (e.g. macrolides, fluoroquinolones, and trimethoprim-sulfamethoxazole) are available but generally less effective [29]. Delayed diagnosis or prolonged disease may require extended and combined antibiotic therapy [21]. Prevention options remain limited, as the only licensed human vaccine (Q-VAX^Ⓡ^, Seqirus Pty Ltd, Australia) is only available in Australia due to adverse effects in previously exposed individuals [30,31]. Efforts to develop safer vaccines are ongoing [31].

Human Q fever incidence varies widely across Europe (Note S2) [32], with sporadic cases dominating in endemic areas and large-scale outbreaks being more likely in nonendemic areas or changing agroecological contexts (e.g. the Dutch outbreak) [[33], [34], [35], [36]]. Outbreaks in nonendemic areas mostly involve clusters of acute cases, whereas in endemic areas, unrelated chronic cases may be detected alongside outbreak-associated acute cases due to ongoing low-level transmission. Long-term follow-up after major outbreaks is essential for early identification and management of chronic cases [37].

Occupationally exposed groups (e.g. veterinarians, slaughterhouse workers, livestock farmers, culling workers, shearers, wool sorters, or field study researchers) show higher *C. burnetii* seroprevalence, but they are not proportionally more clinically affected [[38], [39], [40], [41], [42], [43], [44], [45]], suggesting that repeated exposure may induce a state of immune equilibrium rather than increased vulnerability. Conversely, Q fever also occurs among populations without direct animal contact [[46], [47], [48], [49]], likely due to windborne dispersal of *C. burnetii* from infected farms or slaughterhouses [50]. However, fine-scale source tracking is rarely achieved, notably because genotyping is rarely possible.

This position paper, prepared as part of the European Union (EU)–funded project Q-Net-Assess (https://q-net-assess.com/), seeks to: (a) compare human outbreak detection and notification protocols across six European countries (Belgium, France, Germany, Spain, The Netherlands, and the United Kingdom), (b) examine current practices and challenges in source identification and outbreak mitigation, (c) compile operational experiences for management of human Q fever outbreaks within a One Health framework, and (d) formulate research priorities and recommendations for strengthening outbreak preparedness and integrative risk management.

## Methods

Experts on human Q fever and animal coxiellosis, affiliated to national reference laboratories and healthcare and research institutions in Belgium, France, Germany, Spain, The Netherlands, and the United Kingdom, gathered detailed information on national protocols applied during human Q fever outbreak investigation and management. The inventory included the functions of national reference centres/laboratories (NRCs/NRLs), case definitions, outbreak detection and notification procedures, the methods used to investigate cases and identify the source, and the control interventions implemented. In addition, a narrative review of key Q fever outbreaks in the literature and a structured inventory of scientific articles and public reports detailing outbreak investigations in the consortium countries were compiled to overview outbreak settings, acquisition contexts, suspected sources, and investigations performed. More details on the methodology used are available in Note S3.

## Current practices for the management of human Q fever outbreaks in the six EU consortium countries: Belgium, France, Germany, Spain, The Netherlands, and the United Kingdom

The main characteristics of the human Q fever outbreak investigation and management practices in the six participating countries are presented in Table S1. Briefly, in the six partner countries, there is a designated NRL for animal coxiellosis, but only four have a human Q fever NRC/NRL. The roles of NRCs/NRLs differ between countries, but all provide confirmatory testing upon request. They neither centralize all national diagnoses nor conduct nationwide data collection. In practice, most consortium countries follow the European Centre for Disease Prevention and Control (ECDC) framework for case definition in the context of an outbreak, in which a confirmed acute Q fever case combines compatible clinical signs and at least one positive laboratory test (typically based on serology), whereas a probable case presents clinical signs plus a clear epidemiological link but without laboratory confirmation [51]. The main difference between countries is whether, in the absence of any other positive laboratory result, a single high IgM/IgG titre can be considered laboratory confirmation when clinical criteria and strong epidemiological evidence exist (e.g. France, Germany, and Spain); otherwise, such cases are classified as probable (The Netherlands and the United Kingdom). In Belgium, a patient with high titres of IgM and IgG antibodies in a single sample, even without clinical/epidemiological information, is considered as a confirmed case, thus deviating from the ECDC definition. The United Kingdom is currently reviewing its case definitions.

Q fever is a notifiable disease in humans (acute and chronic) in Belgium and Spain, whereas only acute cases are notifiable in Germany and The Netherlands; in the United Kingdom, all laboratory detections of *C. burnetii* must be reported, and in France, notification is voluntary. Alert criteria vary but generally require at least two epidemiologically linked cases, while some countries act on a single confirmed case, and all raise alerts for unusual increases in severe or atypical Q fever presentations. Reporting timelines and bodies also vary across countries (Table S1).

Once the initial cases are identified, the local public health authority typically conducts the first epidemiological assessments, followed by activation of a multidisciplinary, multiagency outbreak investigation team integrated by public and animal health authorities, veterinarians, epidemiologists, Q fever specialists, and communication specialists. This team (hereinafter referred to as ‘One Health’ team) coordinates further investigations aimed at identifying the source, analysing risk factors, and proposing measures to control and mitigate the outbreak. National‑level coordination may be required depending on the outbreak’s spread and regional capacity, with relevant authorities informed as needed if not already part of the One Health team. In practice, the composition, maturity, and operational capacity of these teams vary across countries, and communication channels are not always well established.

The epidemiological investigation, aimed at establishing timelines, epidemic curves, and maps, is supported by standardized or custom-made epidemiological questionnaires adapted to the specific situation of each outbreak. Collected patient data (age, gender, address, and occupation), case data (date of onset of symptoms, hospitalization, and related patients), and exposure data (proximity to livestock farms, visits to farms, type of pets, whether direct or indirect exposure to animals around parturition, occupational exposure, and travelling abroad) are analysed to identify new cases and to inform decisions regarding the need to investigate potential animal sources. In all consortium countries, information from animal health authorities on *C. burnetii*–infected animal premises, high-risk farming activities, and other potential sources of shedding helps identify nearby ruminant farms as likely sources, with countries applying different search areas (5 km in Belgium and The Netherlands, 8 km in Germany, and variable distances elsewhere). Information on recent abortions and *C. burnetii* presence is collected from these farms, and active sampling may be conducted in coordination with local veterinary authorities when justified and cost-effective. Diagnostic approaches include *C. burnetii*–specific PCR on placenta, vaginal fluids, or bulk-tank milk, combined with serology (e.g. bulk-tank milk and/or sera from nulliparous, primiparous, and multiparous animals). Albeit not routinely performed, environmental dust sampling has been used for research-driven outbreak investigations in France, Spain, and The Netherlands.

Outbreak management depends on the setting, but when farms are suspected or confirmed sources, a range of on-farm control and biosecurity measures (e.g. proper disposal of birthing materials and manure, restricting animal movements and visitors access to housing) are recommended in all consortium countries or are compulsory in some cases (Table S1). Vaccination is compulsory for *C. burnetii*–positive goat herds in Belgium and, in The Netherlands, has been mandatory since 2010 for dairy goat and sheep flocks with more than 50 animals, and for any small‑ruminant farms open to the public or attending exhibitions. In other countries, vaccination is recommended and may become compulsory only when a positive flock/herd is associated with several human cases, with the decision depending on the nature of the farm business and potentially involving assistance from local authorities (e.g. the United Kingdom and Spain). More severe restrictions, including wide-scale animal culling (shedding/pregnant animals) or restrictions on breeding, were exceptionally implemented when the largest outbreak ever reported in the world occurred in The Netherlands in 2005–2012 [52,53].

Most countries recommend heat-treating milk from *C. burnetii*–positive farms, although requirements differ, being compulsory under certain circumstances and periods in Belgium for raw goat milk and raw-milk products, when epidemiologically linked to human outbreaks in The Netherlands, or extend to all zoonotic agents in Germany regardless of human cases. In France, heat treatment is no longer recommended due to the negligible estimated oral transmission risk [54].

In the consortium countries, farmers are trained about the zoonotic risks of *C. burnetii*, whereas high-risk professionals (e.g. veterinarians, slaughterhouse workers, and researchers) receive guidance on infection sources, biosecurity procedures, safe sample handling, and proper use of personal protective equipment.

Q fever outbreaks may also occasionally be related to pets or occur in settings without a clear link to infected livestock (Note S4), and therefore, context-specific source investigation approaches and tailored countermeasures are applied.

## Operational challenges, key gaps in tools and coordination, and recommendations for preparedness and management of human Q fever outbreaks

**Fig. 1:**
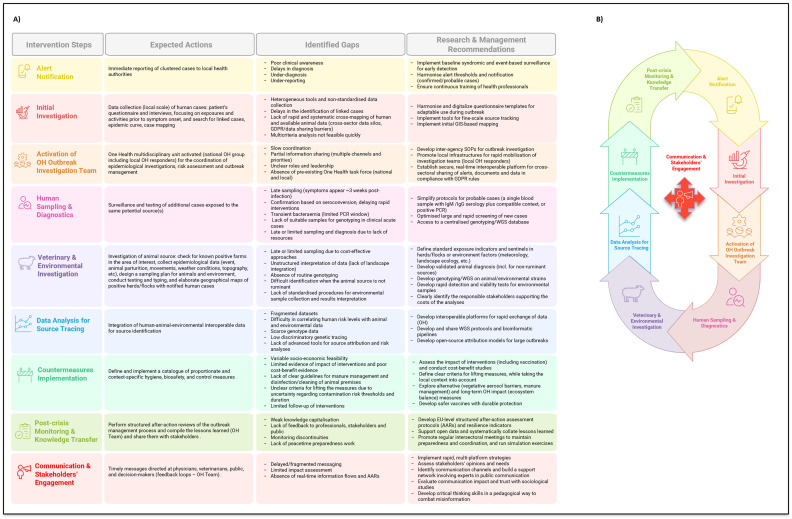
Integrated One Health (OH) framework for Q fever outbreak management. (a) Overview of key operational steps, expected actions, identified operational gaps, and priority actions across the Q fever outbreak response cycle. (b) Schematic representation illustrating the iterative nature of the integrated OH approach to Q fever outbreak management and the central, cross-cutting role of communication. AARs, After-Action Reviews, that is structured, team-based methods of evaluation of an activity to identify what worked, what did not, and why, in order to improve future performance; EU, European Union; GDPR, General Data Protection Regulation; GIS, Geographic Information System; SOP, standard operating procedure; WGS, whole genome sequencing.

The compendium of practices and field experiences for human Q fever outbreak investigation presented here enabled us to map both the convergence and divergence in national approaches across the six European countries, identifying not only key interventions but also persistent operational bottlenecks. The comparison also highlighted critical gaps in diagnostic, epidemiological, and One Health tools and in intersectoral coordination, which warrant further development and harmonization (Fig. 1). Based on the state of the art, actionable recommendations for immediate outbreak response together with priority areas for strengthening long-term preparedness are provided.

### Q fever diagnosis and reporting

Reported human Q fever case numbers and notification rates vary widely across the consortium countries [32], reflecting not only different epidemiological situations but also marked variations in disease awareness, diagnostic laboratory methods and interpretation, case definitions (criteria for confirmed vs. probable cases), and notification protocols (which clinical forms are notifiable, at what level), all of which influence clinical recognition, laboratory confirmation, and how and when cases are formally reported in surveillance systems.

As an example, official notification may be limited by restrictive or inconsistent case definitions. In some countries, case confirmation requires paired serology to demonstrate seroconversion, whereas others accept high antibody levels as sufficient proof when accompanied by clinical signs and an epidemiological link. In practice, otherwise similar patients may be classified as a confirmed case in one country, but only probable case in another, solely due to differences in these criteria. Similarly, chronic cases are not notifiable in all countries, further limiting comparability of national data. Underreporting may also result from suboptimal implementation of the notification system (delays, incomplete reporting by laboratories or practitioners, and limited awareness of notification duties) or from fears of negative economic consequences of notification, such as restrictions on-farm activities, trade bans, effects on tourism, or reduced consumer confidence following official outbreak declarations.

To better detect acute infection, diagnosis should not be based solely on serology; PCR and serology should be performed simultaneously, as their diagnostic windows only partially overlap, and their relative diagnostic performance depends on the timing of sample collection in relation to symptom onset. Complementary approaches, such as real-time syndromic surveillance of hospitalized patients to monitor for clinical syndromes, including lower respiratory tract infections independently of laboratory confirmation, have been shown to improve early detection and may reduce underdiagnosis [55]. In many settings, the dependence on late serological confirmation further delays case recognition and limits timely collection of exposure histories and relevant environmental or animal samples for source investigation. Because health professionals’ knowledge and awareness are key for recognizing and diagnosing Q fever, continuous medical education remains essential [56]. Overall, reinforcing harmonization of diagnostic workflows, from sampling to results interpretation, case definition, and reporting procedures, is a key prerequisite to reduce underdiagnosis and under-reporting and better capture the true disease burden.

### Initial epidemiological investigation and activation of the One Health outbreak investigation team

After the identification of the initial cases, outbreak-specific questionnaires remain critical for case-finding, identification of risk factors, and tracing of potential sources. Delays in mobilization of trained personnel and in access to exposed populations remain a major bottleneck. Targeted communication is central at this stage: alerting clinicians to consider Q fever in differential diagnoses and informing exposed populations are key to timely case-finding. Overall, the quality and timeliness of these first steps largely condition the effectiveness of subsequent outbreak management.

Upon outbreak declaration, comprehensive cross-sectoral One Health approaches are desirable for effective outbreak investigation and control [57]. Examples of interdisciplinary One Health structures or guidelines include The Netherlands’ Zoonoses Structure (integrated human-veterinary risk analysis system) [58], Belgium’s collaboration of Risk Assessment Groups (RAG—human and Risk Assessment Group – Veterinary – Emerging Zoonoses—veterinary) [59,60], and Germany’s Q-GAPS initiative [61], with similar frameworks elsewhere (Table S1). However, such predefined One Health structures and their early, systematic activation remain the exception rather than the rule, and the degree of coordination and practical integration of One Health principles still vary widely across countries and contexts.

Slow coordination, unclear roles and responsibilities, and partial or delayed information exchange between sectors are recurrent weaknesses. These are further compounded by organizational and legal constraints on data sharing, including data protection requirements. Developing interagency standard operating procedures, running simulation exercises, and using secure, real-time data-sharing platforms between human, animal, and environmental health authorities could help address these gaps. Early, clearly mandated One Health coordination is essential to transform initial clinical and epidemiological signals into a coherent, timely outbreak response.

### Outbreak investigation and source tracing

Sources of Q fever outbreaks are primarily investigated by assessing circulation of the bacterium in nearby animal reservoirs, as inhalation of infectious aerosols shed by infected animals is the main transmission route. Outbreak source detection is particularly challenging when classical risk settings (clearly identified animal sources or direct occupational exposure) are absent or unclear. Although Q fever has historically been viewed as a zoonosis associated with rural or livestock-related environments, the increasing number of community outbreaks in urban or periurban areas challenges this perception. In these settings, indirect transmission via environmental contamination, wind, or fomite spread is often suspected, despite no evident animal contact [62]. Risk of infection is highest within a few kilometres of the presumed source, yet cases have been reported at greater distances, likely modulated by wind patterns and other weather conditions [23,63]. Outbreak source investigations therefore rely on integrating human, animal, environmental, and meteorological data [50,64]. Aerosol dynamics, wind patterns, and landscape features that limit airborne dispersion (such as topography and vegetation barriers) [57] should be considered when interpreting epidemiological models and designing mitigation measures.

Timely animal sampling is essential to test hypotheses regarding outbreak source and transmission pathways. PCR of vaginal fluids and birth products from full-term or aborted females (primiparous and multiparous) and serological testing can be complemented with PCR on bulk-tank milk (dairy herds) and, where available, tests of cell-mediated immunity [65,66]. PCR positivity, *C. burnetii*–specific interferon gamma responses, or herd-level seroprevalence above 50% can indicate active infection [66,67]. PCR detection of *C. burnetii* DNA in farm dust has been related to the clinical Q fever status of the herd [13], but the absence of validated interpretative criteria limits its use in outbreak source investigations. In practice, PCR analysis of environmental samples can serve as a first-line screening tool to rapidly investigate holdings georeferenced in the vicinity of the outbreak, helping prioritize farms for detailed animal sampling [68]. This strategy has been incorporated in several epidemiological studies and has proven useful to generate and refine hypothesis about possible source(s) in settings where animals were not directly present [14,49,[69], [70], [71], [72]]. Nonetheless, further studies are needed to standardize procedures for environmental sampling, distinguish true infection sources from background environmental contamination, assess the zoonotic risk posed by PCR-positive dust, and develop rapid, reliable viability tests for *C. burnetii*.

The effectiveness of source investigations is highly dependent on rapid mobilization; suspected farms should be identified and animals sampled promptly. Yet, given the typical 14- to 21-day incubation period, a significant delay between the patient exposure and the field intervention is inevitable. In addition, several obstacles often hamper rapid and comprehensive outbreak investigation. Limited prioritization of Q fever by health authorities, logistical and financial constraints, and concerns about the potential socioeconomic impact on implicated farms often hinder timely and comprehensive animal sampling, limiting the capacity to confirm the source and reduce ongoing transmission. An integrated approach, centred on the animal reservoir but strongly modulated by environmental and landscape drivers, should guide future outbreak management and biosecurity policies, ensuring that they remain proportionate to the level of risk and to the local socioeconomic context.

According to the systematic review by Tan *et al.* [62] and many of the outbreaks reviewed here (Note S4 and Table S2), sheep, goats, and to a lesser extent cattle, are the main sources of human Q fever outbreaks. The reason for the predominance of a sheep link to human outbreaks is unknown; *C. burnetii* virulence factors and genotype may contribute to the marked variation in host range and outcome of infection [27,65,[73], [74], [75], [76]]. However, the full spectrum of potential reservoirs of *C. burnetii* is not fully characterized. The high prevalence in nontraditional hosts, including wildlife species closely interacting with livestock and humans [77], highlights the need to avoid a restrictive and exclusive view. Because data on the actual role of these species, including genotype diversity and zoonotic potential, are limited, future outbreak investigations should remain open to atypical sources, especially when events occur in unusual epidemiological contexts.

Robust source attribution ultimately requires genotyping of *C. burnetii* from human, animal, and ideally environmental samples. Multispacer sequence typing, multiple-locus variable number of tandem repeat analysis, and single-nucleotide polymorphism typing methods can be applied directly to clinical samples without prior culturing and are valuable for epidemiological tracking [[78], [79], [80]]. Whole genome sequencing provides the highest resolution for outbreak investigation and enables full characterization of virulence and host-range determinants, but further developments are needed before it can be directly applicable to complex clinical and environmental matrices without prior culture. In practice, however, human *C. burnetii* strains are rarely available because diagnosis relies mostly on serology, and samples are seldom collected during bacteraemia. Consequently, the absence of systematic genotyping in both humans and animals still limits robust source attribution, which often relies on converging but indirect epidemiological evidence.

Expanding biobanks with *C. burnetii*–positive samples from multiple hosts and locations, and applying comparative genomics, is essential to assess the zoonotic threat of circulating strains, identify spillover drivers, and clarify reservoir roles. Timely field sampling and high-resolution genotyping tools for fine-scale source tracking are needed to accurately determine the relative contribution of different sources and transmission pathways during outbreaks.

### Implementation of countermeasures

The primary aim of outbreak response is to reduce human exposure by limiting animal-to-human spillover. Consortium countries apply similar control measures, but national regulatory approaches vary due to differences in Q fever epidemiology, livestock production systems, and national strategies for managing animal health and zoonotic risk.

Use of protection class filtering face piece 2 respirator masks during outbreak investigations or on-farm interventions is now widely recommended, particularly since the COVID-19 pandemic. However, its effectiveness to prevent inhalation of environmental forms of *C. burnetii* remains unquantified, and further studies are needed to characterize the nature and size of inhaled particles (dust-borne forms) under field conditions.

Current on-farm hygiene and biosecurity recommendations rely mainly on expert consensus rather than robust farm-level evidence. While proper handling of parturition materials and manure, restricted access to animal housing, and cleaning/disinfection are usually recommended, their actual impact reducing environmental contamination and human risk, and the criteria for lifting restrictions remain unclear. In addition, the effectiveness against *C. burnetii* of cleaning/disinfection in the environment of livestock buildings has not been formally validated as the conditions that determine survival or inactivation of *C. burnetii* across complex matrices (dust, manure, slurry, or milk) remain insufficiently characterized. Moreover, such interventions may have unintended adverse effects, including increased aerosolization of contaminated dust or disruption of the natural microbiota, potentially affecting animal health, protective microbial balances, and raw-milk cheese production [81]. Consistent with the One Health approach, countermeasures should also avoid disproportionate impacts on animal health, farm livelihoods, and local ecosystems.

Vaccination against *C. burnetii* is legally regulated for some small ruminant species and/or circumstances in Belgium and The Netherlands but remains a recommendation elsewhere. In The Netherlands, wide-scale animal culling combined with mandatory vaccination of specific groups of sheep and goats (a practice that remains compulsory today) reduced human Q fever cases and lowered bacterial shedding in vaccinated animals [82,83], with subsequent declines in *C. burnetii* detection in bulk-tank milk [84,85]. However, sustained multiyear vaccination is needed to achieve these effects [10,86], adding costs for farmers. Recent observations in The Netherlands [87] indicate that this vaccination strategy may cause side effects that significantly affect the welfare of dairy goats. Besides, the benefit-risk balance of vaccinating infected herds with substantial pre-existing natural immunity remains debated [88]. Systematic cost-effectiveness analyses are scarce.

Recommendations for heat treatment of milk from *C. burnetii*–positive farms are similarly controversial: some authorities consider the infection risk linked to milk consumption negligible [54], whereas others enforce stricter requirements [89]. Given the huge economic impact of this measure, especially for raw-milk cheese producers, further targeted risk assessments and context-specific guidance are needed.

Most countermeasures are not mandatory in the consortium countries, and their effectiveness and specific impact on reducing *C. burnetii* loads are difficult to disentangle from the potential natural decline in prevalence over time, likely associated with increasing herd immunity [57]. Implementation requires willingness and the active participation of veterinarians and farmers, and often faces practical limitations [81] such as insufficient funding, uncertainty about effectiveness, and limited monitoring of the outcomes. Future efforts should prioritize defining the most efficient, context-appropriate combinations of countermeasures and establishing clear criteria for lifting them, alongside developing harmonized yet adaptable protocols. These protocols should aim not only to reduce human exposure in the short term but also to integrate environmental, animal health, and socioeconomic considerations into a coherent One Health control strategy.

### Timely communication and postcrisis knowledge transfer

Effective outbreak management relies on timely, targeted communication with clinicians and public health institutes, farmers, veterinarians, local authorities, and potentially exposed individuals to ensure early awareness, clarify the rationale for restrictions, and facilitate acceptance of control measures. This requires not only biomedical and veterinary expertise but also input from social and behavioural sciences to understand perceptions, trade-offs, and barriers to compliance. Communication should be two-way, allowing feedback from field actors to refine risk assessments and adjust interventions in real time. Clear, consistent messages delivered through appropriate channels are also critical to counter misinformation and avoid unnecessary alarm.

Once the outbreak is under control, the One Health team should review the entire management process. Lessons learned should be compiled and shared with stakeholders to strengthen preparedness, foster cross-sectoral coordination, and improve future response. Whenever possible, the outcomes of these reviews should be formalized into updated standard operating procedures, guidance documents, and training materials, rather than remaining as informal experience. Linking these after-action reviews with continuous surveillance, trend analysis, and regular cross-sectoral meetings can progressively enhance early detection and management of future Q fever outbreaks and other airborne zoonoses.

## Conclusions

Q fever outbreak investigation and control require strong cross-sectoral coordination, harmonized protocols, and sustained resources. Yet surveillance and reporting systems for Q fever across the European countries studied remain heterogeneous, reflecting differences in epidemiology, diagnostics, and public health priorities. In view of the gaps and disparities observed, efforts to harmonize case definitions, diagnostic criteria, and reporting processes—while increasing transparency and awareness among professionals—are urgently needed. A coordinated framework is also necessary to guide the involvement of relevant stakeholders in detection, notification, and response activities. More clearly defined and operational protocols for detecting human Q fever cases, identifying source(s) of infection, and tracing transmission chains would help to design more effective intervention strategies, limit animal-to-human spillover, and reduce the risk of future outbreaks. In the longer term, a more coherent EU framework would enhance data comparability, support robust risk assessment, and strengthen coordinated responses to cross-border threats.

## Declaration of AI and AI-assisted technologies in the writing process

During the preparation of this work the authors used the online visualization tool Napkin AI to support Fig. 1 layout and visual synthesis. In addition, ChatGPT (OpenAI) was used to assist with language editing and improve clarity during manuscript preparation. No data generation, analysis, or interpretation was performed using these tools. After using these tools, the authors reviewed and edited the content as needed and take full responsibility for the content of this publication.

## Funding

This study was funded by ICRAD, an ERA-NET co-funded under European Union's Horizon 2020 research and innovation programme (https://ec.europa.eu/programmes/horizon2020), within the framework of the project ‘Improved molecular surveillance and assessment of host adaptation and virulence of *Coxiella burnetii* in Europe’ (Q-Net-Assess), under Grant Agreement number 862605. UK partners (Moredun Research Institute and Animal and Plant Health Agency) acknowledge funding from the Biotechnology and Biological Sciences Research Council Grant reference: BB/X020142/1. French Partners (ANSES and INRAE) received funding from the Agence Nationale de la Recherche (ANR) under Grant Agreement ‘ANR-22-ICRD-0001-06.’ NEIKER received funding from MICIU/AEI/10.13039/501100011033 under Project PCI2023-143391. Sciensano received funding from the Federal Public Service of Health, Food Chain Safety and Environment contract RI 23/50. Friedrich-Loeffler-Institut received funding from the Federal Ministry of Food and Agriculture under project number 2823ERA30D. Royal GD received funding from the Dutch Ministry of Agriculture, Fisheries, Food Security and Nature.

## CRediT authorship contribution statement

**Ana Hurtado:** Writing – review & editing, Writing – original draft, Visualization, Validation, Supervision, Project administration, Funding acquisition, Formal analysis, Data curation, Conceptualization. **Elodie Rousset:** Writing – review & editing, Writing – original draft, Visualization, Validation, Project administration, Funding acquisition, Formal analysis, Data curation, Conceptualization. **Aurélie Couesnon:** Writing – review & editing, Writing – original draft, Visualization, Validation, Formal analysis, Data curation. **Tara de Haan:** Writing – review & editing, Writing – original draft, Visualization, Validation, Formal analysis, Data curation, Conceptualization. **Frederika Dijkstra:** Writing – review & editing, Validation, Conceptualization. **Silke F. Fischer:** Writing – review & editing, Validation. **Pierre-Edouard Fournier:** Writing – review & editing, Validation. **Ana L. García-Pérez:** Writing – review & editing, Writing – original draft, Visualization, Validation, Formal analysis, Data curation, Conceptualization. **Isabel Jado:** Writing – review & editing, Validation. **Elsa Jourdain:** Writing – review & editing, Writing – original draft, Visualization, Validation, Formal analysis, Data curation, Conceptualization. **Katja Mertens-Scholz:** Writing – review & editing, Writing – original draft, Visualization, Validation, Project administration, Funding acquisition, Formal analysis, Data curation, Conceptualization. **Tom N. McNeilly:** Writing – review & editing, Writing – original draft, Visualization, Validation, Project administration, Funding acquisition, Formal analysis, Data curation, Conceptualization. **Susan Neale:** Writing – review & editing, Writing – original draft, Visualization, Validation, Formal analysis, Data curation, Conceptualization. **Jane C. Osborne:** Writing – review & editing, Validation. **Marjan Van Esbroeck:** Writing – review & editing, Validation. **Marcella Mori:** Writing – review & editing, Writing – original draft, Visualization, Validation, Project administration, Funding acquisition, Formal analysis, Data curation, Conceptualization. **René van den Brom:** Writing – review & editing, Writing – original draft, Visualization, Validation, Project administration, Funding acquisition, Formal analysis, Data curation, Conceptualization.

## Declaration of competing interest

The authors declare that they have no conflicts of interest.

## Appendix Supplementary materials

Image, application 1

Image, application 2

## Footnotes

Supplementary material associated with this article can be found, in the online version, at doi:10.1016/j.cmicom.2026.105195.
