# Supplementary material for: Human Q fever outbreak investigation and management in six European countries: a One Health compendium of practices and recommendations
Source: CMI Commun. 2026 Jul;3(2):None. doi: 10.1016/j.cmicom.2026.105195 (PMC13334506; doi:10.1016/j.cmicom.2026.105195)
Supplement: Supplementary file 1 [file mmc1.pdf]

Supplementary Notes S1-S4

Human Q fever outbreak investigation and management in six European countries: a One Health compendium of practices and recommendations

Ana Hurtado[1*], Elodie Rousset[2], Aurélie Couesnon[2], Tara de Haan[3], Frederika Dijkstra[4], Silke Fischer[5], Pierre-Edouard Fournier[6], Ana L. García-Pérez[1], Isabel Jado[7], Elsa Jourdain[8], Katja Mertens-Scholz[9], Tom N. McNeilly[10], Susan Neale[11], Jane C. Osborne[12], Marjan Van Esbroeck[13], Marcella Mori[14], René van den Brom[3]

[1] Animal Health Department, NEIKER – Basque Institute for Agricultural Research and Development, Basque Research and Technology Alliance (BRTA), Bizkaia Science and Technology Park 812L, 48160 Derio, Spain.

[2] ANSES, Laboratoire de Sophia Antipolis, Unité fièvre Q animale, 06902 Sophia Antipolis, France

[3] Small Ruminant Health Department, Royal GD, Deventer, 7400 AA, The Netherlands

[4] Centre for Infectious Disease Control, Netherlands Institute of Public Health and the Environment (RIVM), Bilthoven, The Netherlands

[5] Landratsamt Rems-Murr-Kreis, Alter Postplatz 18, 71332 Waiblingen, Germany

[6] French reference centre for rickettsioses, Q fever and bartonelloses, IHU Méditerranée Infection, 19-21 Bd Jean Moulin, 13005 Marseille, France

[7] Reference and Research Laboratory on Special Pathogens, National Centre for Microbiology, Instituto de Salud Carlos III (CNM - ISCIII), Madrid, Spain

[8] University of Clermont Auvergne, INRAE, VetAgro Sup, UMR EPIA, Saint-Genès Champanelle, France

[9] Friedrich-Loeffler-Institut, Institute of Bacterial Infections and Zoonoses, Jena, 07743, Germany

[10] Moredun Research Institute, Penicuik, EH26 0PZ, UK

[11] Penrith Veterinary Investigation Centre, Animal and Plant Health Agency (APHA), Cumbria, CA11 9RR, UK

[12] Rare and Imported Pathogens Laboratory, UK Health Security Agency, Porton Down, SP4 0JG, UK

[13] Department of Clinical Sciences, Institute of Tropical Medicine, Antwerp, Belgium

[14] Sciensano, Belgian Institute for Health, Brussels, Belgium

*Corresponding author ahurtado@neiker.eus

Supplementary Note S1 — Human diagnostics background

Laboratory diagnosis of *C. burnetii* infection in humans relies primarily on serology. In acute Q fever, demonstrating seroconversion, or an increase in IgM/IgG antibodies, against phase II in paired serum samples taken at least ten days apart is considered as the best approach. However, in many instances a single sample is provided. In such cases, thresholds can be established (e.g. anti-Phase II IgG titre ≥ 200 and/or anti-Phase II IgM ≥ 50 [1,2]) to support case classification in the absence of another confirmatory test result. Nevertheless, the detection of non-specific IgM antibodies to phase II has been reported as a result of cross-reactivity with other infections (e.g. *Legionella*, *Rickettsia*, *Neisseria*, *Bartonella*, among others), which must be ruled out by differential diagnosis [3]. Furthermore, low IgM antibody levels may persist for a prolonged time,

and therefore a moderate to low IgM titre or ratio does not prove a recent infection [4]. The detection of elevated IgG antibodies to phase I, together with sustained IgG antibodies to phase II in serial serum samples is indicative of chronic infection; in such cases the evaluation of risk factors using echocardiography or other diagnostic imaging tests is recommended [5,6].

Presence of *C. burnetii* in a clinical specimen can be determined by the detection of its nucleic acids (PCR) or antigens (immunochemistry), or by culture. Detection of *C. burnetii* by PCR from blood or serum requires the collection of samples during the transient bacteraemia, which is difficult to achieve, particularly outside of outbreak situations. PCR from blood or serum can nevertheless be used as an early diagnostic test, but the sensitivity is only high before antibody response develops and then declines thereafter [7]. Therefore, PCR should be combined with serology. PCR from respiratory samples may be attempted, but this is often negative in patients with mild clinical symptoms, and the outcome of the test might be influenced by recent consumption of *C. burnetii*-containing products such as milk or cheese [8]. Broncho-alveolar lavage is usually too invasive. In tissue samples, bacterial loads of *C. burnetii* may be high and can be successfully detected by PCR or immunochemistry. However, availability of tissue specimens is most often limited to chronic cases.

Finally, isolation of *C. burnetii* from clinical specimens by culture is not a commonly used diagnostic method due to its obligate intracellular lifestyle and requirements of biosafety level 3 (BSL-3) laboratory facilities. Furthermore, frequent initiation of antibiotic therapy prior to sampling further reduces isolation success. However, isolation is the most suitable method to obtain sufficient bacteria for whole genome characterisation. Alternatively, typing methods can also be applied directly to *C. burnetii*-positive clinical samples without the need for a prior culture step [9–11].

Supplementary Note S2 — Incidence of Q fever in the partner countries: Belgium, France, Germany, Spain, the Netherlands, and the United Kingdom

Q fever surveillance in humans is generally passive, but systems are not harmonised across countries. Human data provided in the EU One Health Zoonoses annual reports, published by the European Food Safety Authority (EFSA) and the European Centre for Disease Prevention and Control (ECDC), are based on case notifications from national health systems, but possible diagnostic and reporting biases cannot be ruled out. Besides, the number of cases reported should be regarded as an underestimation of the true number, as symptoms are non-specific and protocols for disease investigation differ between countries. For example, the diagnosis of Q fever is often not performed routinely in patients with pneumonia in the Netherlands, probably due to the low incidence of the disease or concerns about false-positive results [12]. In France, Q fever is not part of first-line tests performed for pneumonia, but it is frequently investigated when the first-line tests are negative. In regions with a high incidence of Q fever, such as the

Basque Country (Spain), where annual rates are among the highest nationwide [13,14], Q fever is well-known among physicians and considered in the diagnosis of pneumonia when the first-line tests are negative. In Belgium, some hospitals have recently implemented syndromic diagnosis panels using PCR for various respiratory agents including *C. burnetii*. Moreover, in France, where notification is not mandatory, data transmitted to ECDC mainly reflect cases diagnosed at the NRC hospital and are not nationally representative. Therefore, comparisons of incidence data between countries should be made with caution. Nevertheless, the EU One Health annual zoonosis reports compile cross-country human and animal datasets and can be used to describe broad trends in notification patterns. Despite these limitations, they provide the most comprehensive dataset available to date. The analysis on notification rates per 100,000 inhabitants in the consortium countries extracted from these reports (Fig. 1) indicates substantial variation between countries rather than a uniform pattern.

Figure 1. Notification rates per 100,000 inhabitants of confirmed cases in 2014-2023 in Belgium, France, Germany, Spain, the Netherlands, the United Kingdom, and the European Union (EU). Data extracted from the EU One Health annual zoonosis reports prepared by EFSA and ECDC [15,16]. Values for EU include notifications from all Member States except Austria (Q fever is not notifiable and no surveillance system exists) and overseas territories. Spain did not provide information on estimated coverage until 2016, so notification rates cannot be estimated before 2016. Data for the UK is not included in the EU reports after 2019 whilst case definitions and notification pathways are reviewed.

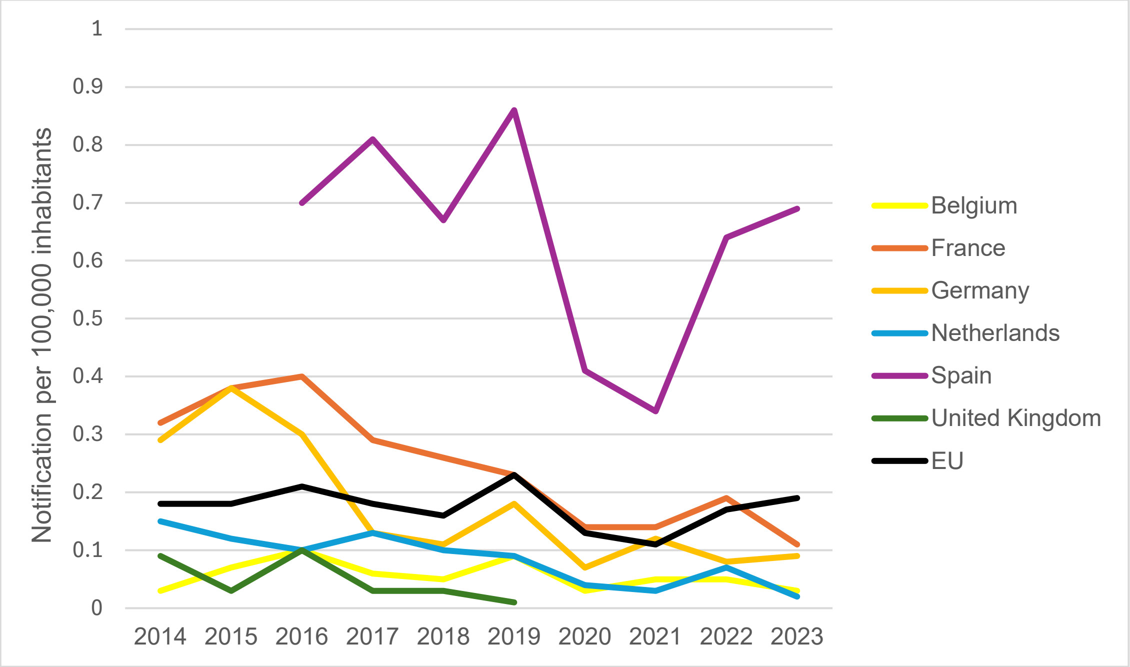

Supplementary Note S3 — Methodology

A **national inventory** on the main components of human Q fever outbreak investigation and management protocols was compiled within the Q-Net-Assess consortium by participating researchers and experts affiliated with academic, public health, veterinary and reference institutions in Belgium, France, Germany, Spain, the Netherlands and the United Kingdom. The aim of the inventory was comparative and descriptive. It was not designed as a formal audit of national systems. The objective was not to rank countries or estimate comparative effectiveness, but to document heterogeneity, identify recurring bottlenecks, and derive a practice oriented One Health framework for outbreak preparedness and management.

To reinforce the structured national inventory of human Q fever outbreak investigation and management practices across the six consortium European countries, we incorporated two complementary components: (i) a **narrative review** of key published articles on human Q fever outbreaks, with particular attention to reports including investigation of animal and/or environmental sources; and, (ii) an **inventory of scientific articles and national reports** available on public institutional websites detailing **human Q fever outbreak investigations** in the consortium countries (Belgium, France, Germany, Spain, the Netherlands and the United Kingdom). The literature component was narrative rather than systematic and was used to provide a broader geographical perspective on key and significant human Q fever outbreak studies that include investigation of the animal/environmental source. This literature component was used to complement the country inventory, identify recurring patterns across outbreak settings, and support the discussion of operational blind spots and research priorities. This was a narrative review rather than a formal systematic review; no PRISMA-based process was applied.

The approach has several limitations. First, the inventory covered six participating countries only and does not constitute a full European mapping. Second, the national outbreak inventory is based on expert input and country-specific documentation rather than on an independent standardised audit. Third, the literature component was narrative and not systematic. Finally, the level of detail available for documented outbreaks varied markedly across settings and countries.

Supplementary Note S4 — Narrative review: Q fever outbreaks in the literature

Q fever has traditionally been considered an occupational disease mainly affecting workers in contact with domestic animals or animal products such as farmers, veterinarians, slaughterhouse and dairy workers, or researchers working with *C. burnetii*. Accordingly, numerous Q fever outbreaks have been reported in settings directly linked to the livestock industry, including slaughterhouses [17–20], ruminant farms [21,22], veterinary hospitals [23–

25], agricultural high schools [26], or animal fairs and markets [27,28]. However, evidence from both the consortium countries and the broader literature indicates that this purely occupational framing of Q-fever risk is now too narrow as Q fever cases and clusters are frequently reported among residents in urban or peri-urban areas without direct contact with farm animals [29–31] or in work settings that are not traditionally considered at risk because they do not typically involve contact with livestock or their products [32–34].

Transmission from animal facilities to non-infected places can occur through contaminated equipment, clothes, or by windborne (aerosol) spread. For example, outbreaks have been associated with the garments or footwear of animal keepers acting as mobile fomites [33,35,36]. The transport or movement of animals may also disseminate *C. burnetii* and lead to infection of drivers, workers, or other people without obvious rural exposure [37–39]. Animal products and/or by-products can further represent risk materials [40]. Similarly, inadequate management of slaughterhouse waste [41] or farm waste such as placentas and manure [32,42–44] have been implicated in several episodes. Also noteworthy is an outbreak suffered by a family after cleaning a pigeon loft [45]. In another outbreak, the most likely cause of spread was through a person coming to work after contact with a litter of newborn cats [46]. Pets, particularly dogs and cats at the time of parturition, have been associated with family or household clusters where infection remained confined to the domestic environment [47–50].

Other less common settings include prisons [51,52], a drug addiction recovery centre [53], psychiatric centres [54,55], or secondary schools [56,57]. In most of these cases, exposure was circumstantially associated with direct contact with animals (used for therapeutic or educational purposes) but also through environmental contamination. Research with pregnant sheep or placenta at university departments has also been associated with outbreaks [58–60]. Rare but illustrative outbreaks include one associated with the use of cell therapy using sheep foetal cells [61] or one in a cosmetics factory working with ovine placentas [62].

The outbreaks documented in the consortium countries are compiled in Supplementary Table S2, along with details on the setting, acquisition context, suspected source, and the level of investigation performed. In Belgium, the largest outbreak involved nine students attending a summer camp in Tel Aviv in 2005 where they—among a total of 117 patients—were exposed to an infected parturient cat [63]. In France, 104 human cases of Q fever were diagnosed over a period of four months (June-September 2002) in association with sheep transhumance in the Chamonix valley [64]. In Germany, *C. burnetii* infected sheep grazing and lambing on a meadow bordering a residential area caused a large Q fever outbreak with 331 cases in 2005 [65,66]; risk of infection increased for residents living closer to the meadow. The largest outbreak recorded in the UK occurred in a co-located slaughterhouse and meat processing plant in Scotland in 2006 and involved 142 cases; airborne transmission from the sheep pre-slaughtering holding area was the most likely cause [19]. In Spain, the largest Q fever outbreak reported affected 108 visitors to a natural cave in 2020-2021, where goats sheltering inside when parturitions occur in winter months, were considered the most likely source of infection [67]. Finally, the largest Q fever outbreak described worldwide, occurred in the Netherlands between 2005 and 2012, with more than 4,000 cases linked to dairy goat farms [68,69]. The epidemic illustrated how rapid changes

in animal husbandry, landscape, and surveillance can trigger explosive transmission and highlighted the substantial public health impact of Q fever.

Across the outbreaks compiled in Supplementary Table S2, infections acquired in the community clearly predominate over events strictly confined to traditional at-risk occupations. Many outbreaks affected residents living near livestock holdings or visiting farms open to the public, while several others arose in unexpected interfaces such as waste-sorting plants, a machine-tool factory, a cardboard manufacturing plant, a pet courier service, or a natural cave used as a tourist attraction. Notably, multiple outbreaks in three countries were linked to visits to farms or farm-like settings open to visitors, highlighting the risk posed by recreational or educational contact with ruminants. In most community outbreaks, the available evidence pointed towards indirect transmission (via environmental contamination, windborne spread or fomites), whereas transmission by direct contact with animals or their tissues was more likely for traditional occupational acquisition.

Where a likely animal source was reported, sheep were most frequently implicated, with goats less often and cattle only occasionally (Supplementary Table S2). Pets, particularly cats and dogs around parturition, were associated with smaller family-based clusters. However, in the majority of episodes, this attribution relied on epidemiological links (exposure histories, spatial patterns, timing of kidding) rather than microbiological confirmation. Truly integrated One Health investigations combining human clinical data with animal and environmental sampling and, in some instances, *C. burnetii* genotyping were performed in only a limited subset of recent outbreaks.

References

[1] Dupont HT, Thirion X, Raoult D. Q fever serology: Cutoff determination for microimmunofluorescence. Clin Diagn Lab Immunol 1994;1:189–96. https://doi.org/10.1128/cdli.1.2.189-196.1994.

[2] Hunfeld K-P. MIQ Heft: 35a Infektionsimmunologische Methoden Teil 1., ed. Mikrobiologisch-infektiologische Qualitätsstandards (MIQ). München: Urban & Fischer Verlag/Elsevier GmbH; 2016.

[3] Altdorfer A, Pirotte BF, Gaspard L, Gregoire E, Firre E, Moerman F, et al. Infective endocarditis caused by *Neisseria mucosa* on a prosthetic pulmonary valve with false positive serology for *Coxiella burnetii* – The first described case. IDCases 2021;24. https://doi.org/10.1016/j.idcr.2021.e01146.

[4] Wielders CCH, Teunis PFM, Hermans MHA, van der Hoek W, Schneeberger PM. Kinetics of antibody response to *Coxiella burnetii* infection (Q fever): Estimation of the seroresponse onset from antibody levels. Epidemics 2015;13:37–43. https://doi.org/10.1016/j.epidem.2015.07.001.

[5] Kampschreur LM, Wegdam-Blans MCA, Wever PC, Renders NHM, Delsing CE, Sprong T, et al. Chronic Q fever diagnosis—consensus guideline *versus* expert

opinion. Emerg Infect Dis 2015;21:1183–8. https://doi.org/10.3201/eid2107.130955.

[6] Wegdam-Blans MCA, Kampschreur LM, Delsing CE, Bleeker-Rovers CP, Sprong T, van Kasteren MEE, et al. Chronic Q fever: Review of the literature and a proposal of new diagnostic criteria. Journal of Infection 2012;64:247–59. https://doi.org/10.1016/j.jinf.2011.12.014.

[7] Schneeberger PM, Hermans MHA, Van Hannen EJ, Schellekens JJA, Leenders ACAP, Wever PC. Real-time PCR with serum samples is indispensable for early diagnosis of acute Q fever. Clinical and Vaccine Immunology 2010;17:286–90. https://doi.org/10.1128/CVI.00454-09.

[8] Buijs SB, Hermans MHA, Agni N, de Vries MC, Hoepelman AIM, Oosterheert JJ, et al. Pitfalls of molecular diagnostic testing for *Coxiella burnetii* DNA on throat swabs. J Microbiol Methods 2019;162:16–20. https://doi.org/10.1016/J.MIMET.2019.05.006.

[9] Huijsmans CJJ, Schellekens JJA, Wever PC, Toman R, Savelkoul PHM, Janse I, et al. Single-nucleotide-polymorphism genotyping of *Coxiella burnetii* during a Q fever outbreak in the Netherlands. Appl Environ Microbiol 2011;77:2051–7. https://doi.org/10.1128/AEM.02293-10.

[10] Glazunova O, Roux V, Freylikman O, Sekeyova Z, Fournous G, Tyczka J, et al. *Coxiella burnetii* genotyping. Emerg Infect Dis 2005;11:1211–7. https://doi.org/10.3201/eid1108.041354.

[11] Karlsson E, Macellaro A, Byström M, Forsman M, Frangoulidis D, Janse I, et al. Eight new genomes and synthetic controls increase the accessibility of rapid melt-MAMA SNP typing of *Coxiella burnetii*. PLoS One 2014;9:e85417. https://doi.org/10.1371/journal.pone.0085417.

[12] Rietveld A, de Groot M, Nieman A-E. Q-Koorts PCR op respiratoir materiaal van lage diagnostische waarde voor Q-koortspneumonie / IB 12-2020. Infectieziekten Bulletin, Jaargang 31, Nummer 3 2020.

[13] Cifo D, Estévez-Reboredo RM, González-Barrio D, Jado I, Gómez-Barroso D. Epidemiology of Q fever in humans in four selected regions, Spain, 2016 to 2022. Eurosurveillance 2024;29:2300688. https://doi.org/10.2807/1560-7917.ES.2024.29.27.2300688.

[14] Alende-Castro V, Macía-Rodríguez C, Novo-Veleiro I, García-Fernández X, Treviño-Castellano M, Rodríguez-Fernández S, et al. Q fever in Spain: Description of a new series, and systematic review. PLoS Negl Trop Dis 2018;12:e0006338. https://doi.org/10.1371/journal.pntd.0006338.

[15] EFSA, ECDC. The European Union One Health 2018 Zoonoses Report. EFSA J 2019;17:5926. https://doi.org/10.2903/j.efsa.2019.5926.

[16] EFSA, ECDC. The European Union One Health 2022 Zoonoses Report. EFSA J 2023;21:e8442. https://doi.org/10.2903/j.efsa.2023.8442.

[17] Tonge JI, Kennedy JM. An outbreak of Q fever in an abattoir near Brisbane. Med J Aust 1963;50 (1):340–3. https://doi.org/10.5694/j.1326-5377.1963.tb23064.x.

[18] Gilroy N, Formica N, Beers M, Egan A, Conaty S, Marmion B. Abattoir-associated Q fever: a Q fever outbreak during a Q fever vaccination program. Aust N Z J Public Health 2001;25:362–7. https://doi.org/10.1111/j.1467-842X.2001.tb00595.x.

[19] Wilson LE, Couper S, Prempeh H, Young D, Pollock KGJ, Stewart WC, et al. Investigation of a Q fever outbreak in a Scottish co-located slaughterhouse and cutting plant. Zoonoses Public Health 2010;57:493–8. https://doi.org/10.1111/j.1863-2378.2009.01251.x.

[20] Lord H, Fletcher-Lartey S, Weerasinghe G, Chandra M, Egana N, Schembri N, et al. A Q fever cluster among workers at an abattoir in south-western Sydney, Australia, 2015. Western Pacific Surveillance and Response Journal 2016;7:21–7. https://doi.org/10.5365/WPSAR.2016.7.2.012.

[21] Bjork A, Marsden-Haug N, Nett RJ, Kersh GJ, Nicholson W, Gibson D, et al. First reported multistate human Q fever outbreak in the United States, 2011. Vector-Borne and Zoonotic Diseases 2014;14:111–7. https://doi.org/10.1089/vbz.2012.1202.

[22] Bond KA, Vincent G, Wilks CR, Franklin L, Sutton B, Stenos J, et al. One Health approach to controlling a Q fever outbreak on an Australian goat farm. Epidemiol Infect 2016;144:1129–41. https://doi.org/10.1017/S0950268815002368.

[23] Komiya T, Toriniwa H, Sadamasu K, Fukushi H, Hirai K, Arashima Y, et al. Epidemiological survey on the route of *Coxiella burnetii* infection in an animal hospital. Journal of Infection and Chemotherapy 2003;9:151–5. https://doi.org/10.1007/s10156-003-0237-7.

[24] Kopecny L, Bosward KL, Shapiro A, Norris JM. Investigating *Coxiella burnetii* infection in a breeding cattery at the centre of a Q fever outbreak. J Feline Med Surg 2013;15:1037–45. https://doi.org/10.1177/1098612X13487360.

[25] Malo JA, Colbran C, Young M, Vasant B, Jarvinen K, Viney K, et al. An outbreak of Q fever associated with parturient cat exposure at an animal refuge and veterinary clinic in southeast Queensland. Aust N Z J Public Health 2018;42:451–5. https://doi.org/10.1111/1753-6405.12784.

[26] Rousset E, Raptopoulo A, Poivre M, Lafon J, Thiéry R, Couesnon A, et al. Epidemiological investigation on a dairy sheep farm in a professional agricultural high school following an alert of Q fever clustered human cases. ESCCAR International Congress on Rickettsiae and 9th Meeting of the European Society for *Chlamydia* Research (ESCR) 2022.

[27] Porten K, Rissland J, Tigges A, Broll S, Hopp W, Lunemann M, et al. A super-spreading ewe infects hundreds with Q fever at a farmers' market in Germany. BMC Infect Dis 2006;6:147. https://doi.org/10.1186/1471-2334-6-147.

[28] O'Connor BA, Tribe IG, Givney R. A windy day in a sheep saleyard: an outbreak of Q fever in rural South Australia. Epidemiol Infect 2015;143:391–8. https://doi.org/10.1017/S0950268814001083.

[29] McQuiston JH, Gibbons R V, Velic R, Nicholson WL, Castrodale L, Wainright SH, et al. Investigation of a focus of Q fever in a nonfarming population in the Federation of Bosnia and Herzegovina. Ann N Y Acad Sci 2003;990:229–32. https://doi.org/10.1111/j.1749-6632.2003.tb07368.x.

[30] Tissot-Dupont H, Amadei MA, Nezri M, Raoult D. Wind in november, Q fever in december. Emerg Infect Dis 2004;10:1264–9. https://doi.org/10.3201/eid1007.030724.

[31] Huang M, Ma J, Jiao J, Li C, Chen L, Zhu Z, et al. The epidemic of Q fever in 2018 to 2019 in Zhuhai city of China determined by metagenomic next-generation sequencing. PLoS Negl Trop Dis 2021;15:e0009520. https://doi.org/10.1371/journal.pntd.0009520.

[32] Alonso E, Lopez-Etxaniz I, Hurtado A, Liendo P, Urbaneja F, Aspiritxaga I, et al. Q fever outbreak among workers at a waste-sorting plant. PLoS One 2015;10:e0138817. https://doi.org/10.1371/journal.pone.0138817.

[33] Hurtado A, Alonso E, Aspiritxaga I, López Etxaniz I, Ocabo B, Barandika JF, et al. Environmental sampling coupled with real-time PCR and genotyping to investigate the source of a Q fever outbreak in a work setting. Epidemiol Infect 2017;145:1834–42. https://doi.org/10.1017/S0950268817000796.

[34] van Noten H, Mori M, Morissens M, Maillart E, Leemans S, Gvinda D, et al. A zoonotic cause of blood culture-negative infective endocarditis in Belgium: Case report and review of the literature on Q fever. IDCases 2022;29:e01595. https://doi.org/10.1016/j.idcr.2022.e01595.

[35] Marrie TJ, Langille D, Papukna V, Yates L. Truckin' pneumonia—an outbreak of Q fever in a truck repair plant probably due to aerosols from clothing contaminated by contact with newborn kittens. Epidemiol Infect 1989;102:119–27. https://doi.org/10.1017/S0950268800029757.

[36] Varga V. An explosive outbreak of Q-fever in Jedl'ové Kostol'any, Slovakia. Cent Eur J Public Health 1997;5:180–2.

[37] Dupuis G, Petite J, Péter O, Vouilloz M. An important outbreak of human Q fever in a Swiss Alpine Valley. Int J Epidemiol 1987;16:282–7. https://doi.org/10.1093/ije/16.2.282.

[38] Selvaggi TM, Rezza G, Scagnelli M, Rigoli R, Rassu M, De Lalla F, et al. Investigation of a Q-fever outbreak in Northern Italy. Eur J Epidemiol 1996;12:403–8.

[39] Alonso E, Eizaguirre D, Lopez-Etxaniz I, Olaizola JI, Ocabo B, Barandika JF, et al. A Q fever outbreak associated to courier transport of pets. PLoS One 2019;14:e0225605. https://doi.org/10.1371/journal.pone.0225605.

[40] Shepard CC. An outbreak of Q fever in a Chicago packing house. Am J Epidemiol 1947;46:185–92. https://doi.org/10.1093/oxfordjournals.aje.a119162.

[41] Carrieri MP, Tissot-Dupont H, Rey D, Brousse P, Renard H, Obadia Y, et al. Investigation of a slaughterhouse-related outbreak of Q fever in the French Alps. European Journal of Clinical Microbiology and Infectious Diseases 2002;21:17–21. https://doi.org/10.1007/s10096-001-0645-5.

[42] Harvey MS, Forbes GB, Marmion BP. An outbreak of Q fever in East Kent. The Lancet 1951;258:1152–7. https://doi.org/10.1016/S0140-6736(51)93157-1.

[43] Winner SJ, Eglin RP, Moore VIM, Mayon-White RT. An outbreak of Q fever affecting postal workers in Oxfordshire. Journal of Infection 1987;14:255–61. https://doi.org/10.1016/S0163-4453(87)93560-2.

[44] Berri M, Rousset E, Champion JL, Arricau-Bouvery N, Russo P, Pepin M, et al. Ovine manure used as a garden fertiliser as a suspected source of human Q fever. Veterinary Record 2003;153:269–70. https://doi.org/10.1136/vr.153.9.269.

[45] Stein A, Raoult D. Pigeon pneumonia in Provence: a bird-borne Q fever outbreak. Clinical Infectious Diseases 1999;29:617–20. https://doi.org/10.1086/598643.

[46] Marrie TJ, MacDonald A, Durant H, Yates L, McCormick L. An outbreak of Q fever probably due to contact with a parturient Cat. Chest 1988;93:98–103. https://doi.org/10.1378/chest.93.1.98.

[47] Bauer BU, Knittler MR, Herms TL, Frangoulidis D, Matthiesen S, Tappe D, et al. Multispecies Q fever outbreak in a mixed dairy goat and cattle farm based on a new bovine-associated genotype of *Coxiella burnetii*. Vet Sci 2021;8:252. https://doi.org/10.3390/vetsci8110252.

[48] Pinsky RL, Fishbein DB, Greene CR, Gensheimer KF. An outbreak of cat-associated Q fever in the United States. J Infect Dis 1991;164:202–4. https://doi.org/10.1093/infdis/164.1.202.

[49] Buhariwalla F, Cann B, Marrie TJ. A dog-related outbreak of Q fever. Clinical Infectious Diseases 1996;23:753–5. https://doi.org/10.1093/clinids/23.4.753.

[50] García de Cruz S, Aldea Mansilla C, Nebreda T, Campos Á. Brote familiar de fiebre Q. Enferm Infecc Microbiol Clin 2010;28:326–7. https://doi.org/10.1016/j.eimc.2009.04.014.

[51] Brown GL, Colwell DC, Hooper WL. An outbreak of Q fever in Staffordshire. Journal of Hygiene 1968;66:649–55. https://doi.org/10.1017/S0022172400028382.

[52] Starnini G, Caccamo F, Farchi F, Babudieri S, Brunetti B, Rezza G. An outbreak of Q fever in a prison in Italy. Epidemiol Infect 2005;133:377–80. https://doi.org/10.1017/S0950268804003383.

[53] Boschini A, Di Perri G, Legnani D, Fabbri P, Ballarini P, Zucconi R, et al. Consecutive epidemics of Q fever in a residential facility for drug abusers: Impact on persons with human immunodeficiency virus infection. Clinical Infectious Diseases 1999;28:866–72. https://doi.org/10.1086/515192.

[54] Fishbein DB, Raoult D. A cluster of *Coxiella burnetii* infections associated with exposure to vaccinated goats and their unpasteurized dairy products. Am J Trop Med Hyg 1992;47:35–40. https://doi.org/10.4269/ajtmh.1992.47.35.

[55] Koene RPM, Schimmer B, Rensen H, Biesheuvel M, De Bruin A, Lohuis A, et al. A Q fever outbreak in a psychiatric care institution in The Netherlands. Epidemiol Infect 2011;139:13–8. https://doi.org/10.1017/S095026881000021X.

[56] Jorm LR, Lightfoot NF, Morgan KL. An epidemiological study of an outbreak of Q fever in a secondary school. Epidemiol Infect 1990;104:467–77. https://doi.org/10.1017/S0950268800047476.

[57] Amitai Z, Bromberg M, Bernstein M, Raveh D, Keysary A, David D, et al. A large Q fever outbreak in an urban school in central Israel. Clinical Infectious Diseases 2010;50:1433–8. https://doi.org/10.1086/652442.

[58] Meiklejohn G, Reimer LG, Graves PS, Helmick C. Cryptic epidemic of Q fever in a medical school. J Infect Dis 1981;144:107–13. https://doi.org/10.1093/infdis/144.2.107.

[59] Hamadeh GN, Turner BW, Trible WJr, Hoffmann BJ, Anderson RM. Laboratory outbreak of Q fever. J Fam Pract 1992;35:683–5.

[60] Hall CJ, Richmond SJ, Caul EO, Pearce NH, Silver IA. Laboratory outbreak of Q fever acquired from sheep. The Lancet 1982;319:1004–6. https://doi.org/10.1016/S0140-6736(82)92001-3.

[61] Robyn MP, Newman AP, Amato M, Walawander M, Kothe C, Nerone JD, et al. Q fever outbreak among travelers to Germany associated with live cell therapy — United States and Canada, 2014: a co-publication. Canada Communicable Disease Report 2015;41:223–6. https://doi.org/10.14745/ccdr.v41i10a01.

[62] Wade AJ, Cheng AC, Athan E, Molloy JL, Harris OC, Stenos J, et al. Q fever outbreak at a cosmetics supply factory. Clinical Infectious Diseases 2006;42:e50–2. https://doi.org/10.1086/501127.

[63] De Schrijver K, Gutfreund G, Esbroeck M Van. Q-koorts bij Antwerpse studenten na een verblijf in Israël. 2006.

[64] Rey S, Dennetiere G, Rousset E, Aubert M, Struggar S, Languille J, et al. Epidémie de fièvre Q dans la vallée de Chamonix (Haute-Savoie), juin-septembre 2002. 2005.

[65] Gilsdorf A, Kroh C, Grimm S, Jensen E, Wagner-Wiening C, Alpers K. Large Q fever outbreak due to sheep farming near residential areas, Germany, 2005. Epidemiol Infect 2008;136:1084–7. https://doi.org/10.1017/S0950268807009533.

[66] Boden K, Brasche S, Straube E, Bischof W. Specific risk factors for contracting Q fever: Lessons from the outbreak Jena. Int J Hyg Environ Health 2014;217:110–5. https://doi.org/https://doi.org/10.1016/j.ijheh.2013.04.004.

[67] Hurtado A, Zendoia II, Alonso E, Beraza X, Bidaurrazaga J, Ocabo B, et al. A Q fever outbreak among visitors to a natural cave, Bizkaia, Spain, December 2020 to

October 2021. Eurosurveillance 2023;28:pii=2200824. https://doi.org/10.2807/1560-7917.ES.2023.28.28.2200824.

[68] Roest HIJ, Bossers A, van Zijderveld FG, Rebel JML. Clinical microbiology of *Coxiella burnetii* and relevant aspects for the diagnosis and control of the zoonotic disease Q fever. Veterinary Quarterly 2013;33:148–60. https://doi.org/10.1080/01652176.2013.843809.

[69] van der Hoek W, Morroy G, Renders NHM, Wever PC, Hermans MHA, Leenders ACAP, et al. Epidemic Q fever in humans in the Netherlands. In: Toman R, Heinzen RA, Samuel JE, Mege J-L, editors. *Coxiella burnetii*: Recent advances and new perspectives in research of the Q fever bacterium, Dordrecht: Springer Netherlands; 2012, p. 329–64. https://doi.org/10.1007/978-94-007-4315-1_17.
